# PDZ Domain Recognition: Insight from Human Tax-Interacting Protein 1 (TIP-1) Interaction with Target Proteins

**DOI:** 10.3390/biology4010088

**Published:** 2015-02-05

**Authors:** Smita Mohanty, Mohiuddin Ovee, Monimoy Banerjee

**Affiliations:** Department of Chemistry, Oklahoma State University, Stillwater, OK 74078, USA; E-Mails: mohiuddin.ovee@okstate.edu (M.O.); monimoy.banerjee@stjude.org (M.B.)

**Keywords:** PDZ, TIP-1, GIP, NMR, structure, function, peptide motif

## Abstract

Cellular signaling is primarily directed via protein-protein interactions. PDZ (PSD-95/Discs large/ZO-1 homologous) domains are well known protein-protein interaction modules involved in various key signaling pathways. Human Tax-interacting protein 1 (TIP-1), also known as glutaminase interaction protein (GIP), is a Class I PDZ domain protein that recognizes the consensus binding motif X-S/T-X-V/I/L-COOH of the C-terminus of its target proteins. We recently reported that TIP-1 not only interacts via the C-terminus of its target partner proteins but also recognizes an internal motif defined by the consensus sequence S/T-X-V/L-D in the target protein. Identification of new target partners containing either a C-terminal or internal recognition motif has rapidly expanded the TIP-1 protein interaction network. TIP-1 being composed solely of a single PDZ domain is unique among PDZ containing proteins. Since it is involved in many important signaling pathways, it is a possible target for drug design. In this mini review, we have discussed human TIP-1, its structure, mechanism of function, its interactions with target proteins containing different recognition motifs, and its involvement in human diseases. Understanding the molecular mechanisms of TIP-1 interactions with distinct target partners and their role in human diseases will be useful for designing novel therapeutics.

## 1. PDZ Domains

Proteins containing PDZ (PSD-95/Discs large/ZO-1 homologous) domain, an important protein-protein interaction module [1,2], are predominantly found in eukaryotic organisms [3] but are also found in some prokaryotes. These small interaction modules are present in hundreds of human proteins [4]. These domains normally span 80–100 amino acid residues and are comprised of six β-sheets and two α-helices. A related type of PDZ domain has also been identified in yeast, bacteria and plants [5,6]. Generally, larger proteins are composed entirely of multiple PDZ domains or in combination with other domains such as SH2, SH3 or PH. An important characteristic of most PDZ domains is their recognition of a specific C-terminal sequence motif present in their target proteins. Internal motif recognition by PDZ domains is rare but has been reported in few cases [7,8,9,10]. PDZ domains are classified based on the C-terminus recognition sequence motif of their target proteins. The major classes are: Class I (X-S/T-X-Φ-COOH), Class II (X-Φ-X-Φ-COOH), Class III (X-E/D-X-Φ-COOH), and various other minor classes, where Φ represents any hydrophobic amino acid residue and X is any residue [11,12,13,14,15]. The amino acid residues present at positions 0 and −2 of the peptide (where position 0 denotes the extreme C-terminal residue) play important roles in the specificity and affinity of the interaction [11,16].

Most PDZ domain-containing proteins have more than one PDZ domains [3]. Often PDZ domains interact with other PDZ domains within the same protein or from other proteins [17,18,19,20,21]. Interaction of PDZ domains within a protein could result in structural stability of the functional domain [19] or synergy in ligand binding [20].

PDZ domain-containing proteins are involved in key pathways in normal cells, as well as in diseased states [22]. PDZ domains regulate essential functions such as the clustering of ion channels, protein targeting, membrane expression of receptors, cell polarity, and cell-cell communications [23,24,25,26]. Because PDZ-containing proteins interact with many proteins within a cell and are involved in several diseases [27,28,29], investigation of the mechanisms of PDZ domain-mediated protein-protein interactions is important for understanding the biological functions of PDZ domains and consequently for the possible design of new therapeutics.

## 2. The PDZ Domain of TIP-1

Human Tax-interacting protein 1 (TIP-1), also known as glutaminase interacting protein (GIP) or Tax1-binding protein 3 (Tax1BP3), consists solely of a single PDZ domain encompassing residues 13–112 of the 124-amino acid protein [30]. TIP-1 is an important PDZ protein in the mammalian brain present in both astrocytes and neurons [31]. TIP-1 belongs to the Class I PDZ domain family that recognizes proteins containing an X-S/T-X-I/L/V-COOH C-terminal recognition motif. The list of proteins now known to be recognized by TIP-1 through their C-terminal motif has been steadily growing [16,32,33,34,35]. These interacting partners are involved in various biological functions such as cell proliferation, stress response, development and cell polarization [36,37,38], implicating the role of TIP-1 in the regulation of these processes. Recently, TIP-1 was also reported to recognize some target proteins by binding a novel internal motif [39]. The identification of this alternative mode of recognition has helped in the discovery of new target proteins, suggesting further signaling cascades that may be regulated by TIP-1 via these interactions. Thus, these TIP-1 regulated signaling cascades represent an exciting field of research with the potential of understanding various cellular processes and specific involvement of TIP-1 in such processes.

## 3. PDZ Domain Structure and Mechanism of Ligand Binding

**Figure 1:**
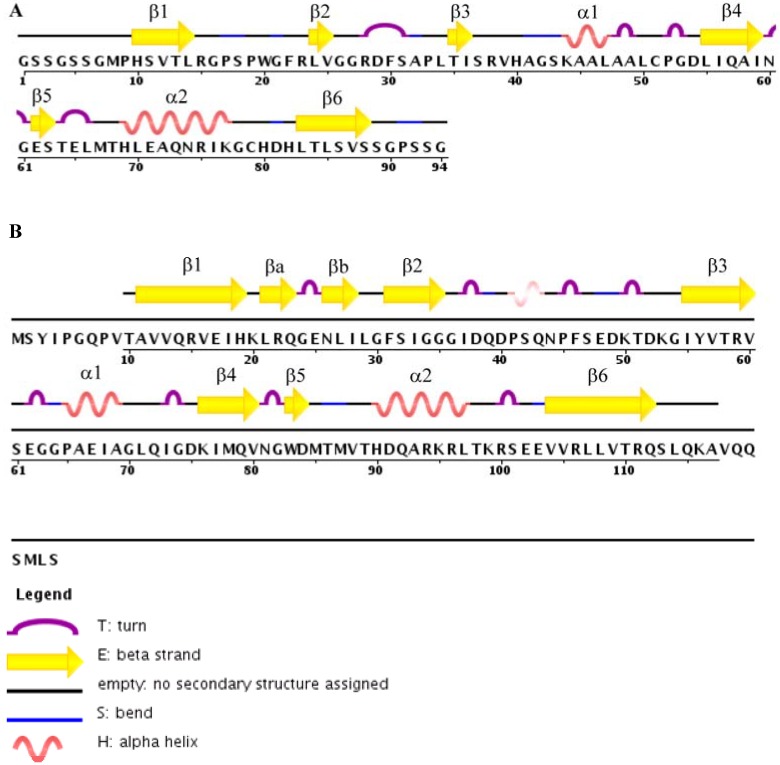
Comparison of structural features of TIP-1 to those of canonical PDZ domain. (**A**) DSSP assignment of the secondary structure corresponding to its amino acid sequence of a canonical PDZ domain (PDB ID: 2EEG). DSSP is the standard method of secondary structure assignment of the amino acid residues of a protein from the atomic-resolution coordinates of the protein. (**B**) DSSP assignment of the secondary structure of TIP-1 (PDB ID: 3GJ9) [45].

The structure of a canonical PDZ domain is characterized by a six-stranded antiparallel β-barrel flanked by two α-helices [3,40] (Figure 1). PDZ domains bind to the C-terminus of target partner proteins in the elongated groove located between the α2-helix and β2-strand, known as the PDZ-binding groove [40,41,42,43]. A Leu-Gly-Phe (LGF) motif, frequently preceded by a Gly residue but alternatively by a Ser, Thr, Ile, or Phe residue [3], is conserved among PDZ-containing proteins. This motif is important for the formation of critical hydrogen bonds to the C-terminal carboxylate group (COO-) of the binding partner [40,44]. The Leu and Phe residues in the highly conserved LGF sequence contribute hydrophobicity to the binding pocket for canonical PDZ domain, while the Gly between them is structurally required to form the proper pattern of hydrogen bonds between the C-terminal carboxylate of the ligand and the protein backbone amides. Such sequence and structural arrangements enable the binding pocket to be correctly oriented for binding of the target protein C-terminal carboxylate [3,40].

## 4. Structures of TIP-1 and TIP-1-Ligand Complexes

**Figure 2:**
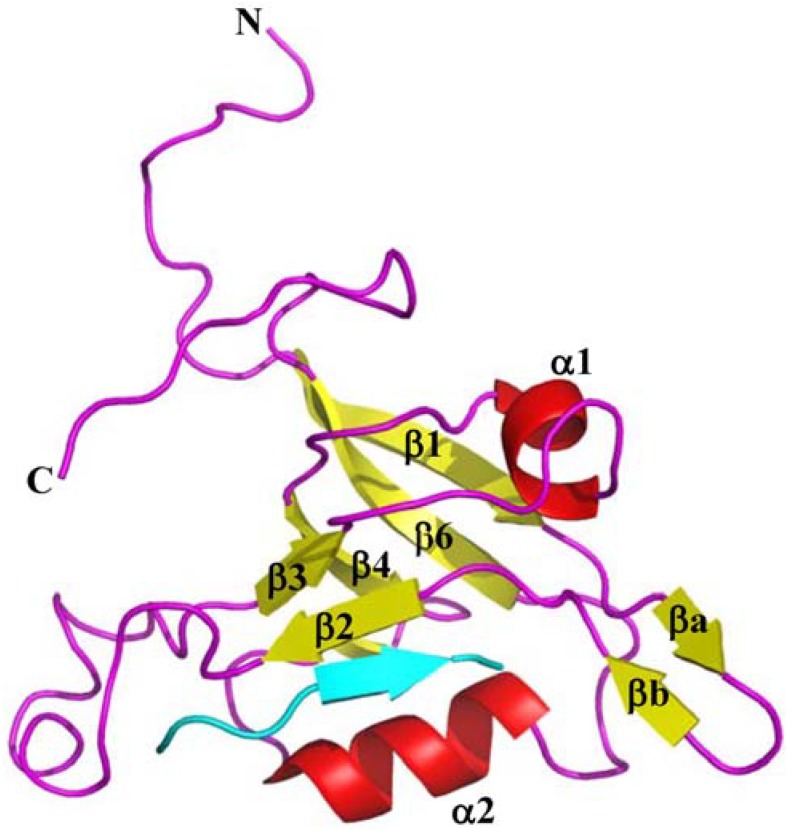
Ribbon diagram of the TIP-1-glutaminase L peptide complex structure. Complexed TIP-1 (Helix: red, Sheet: yellow, Loop: magenta) and the glutaminase L peptide in cyan (PDB code: 2L4T). The figure was generated by Pymol software.

The three-dimensional structures of TIP-1 and TIP-1 in complex with C-terminal peptide analogs of partner proteins have been determined by NMR or X-ray crystallography [45,46,47,48,49] (Table 1). TIP-1 binds to its ligands with an affinity in the low micro-molar range indicating moderate binding affinity [50]. Experimentally determined structures of TIP-1 bound to the peptide analogs representing glutaminase L (KENLESMV-COOH), β-catenin (NQLAWFDTDL-COOH), and Kir 2.3 (RRESAI-COOH) reveal the mechanisms of the interactions between TIP-1 PDZ domain and the above partner proteins [45,47,49]. Structures of TIP-1 in complex with CAL (the cystic fibrosis (CF) transmembrane conductance regulator (CFTR)-associated ligand) inhibitor peptides iCAL36 (ANSRWPTSII) and iCAL36_L_ (ANSRWPTSIL) have also recently been determined [51,52]. All the above TIP-1 target proteins contain a hydrophobic residue (Val or Leu or Ile) as the terminal residue in their C-terminus. The 3D NMR structures of TIP-1 and TIP-1-glutaminase L complex were determined in our laboratory (Figure 2) [49], while TIP-1, TIP-1 bound to β-catenin and Kir 2.3 structures were solved by X-ray crystallography [45,47]. Both the NMR and X-ray structures show that TIP-1 maintains an overall similar topology in both free and bound states with subtle structural changes upon ligand binding [49]. TIP-1 has both similarities and differences with canonical PDZ domain. Like other PDZ domains, TIP-1 has an architecture consisting of six-stranded antiparallel β-barrel flanked by two α-helices, however, it also contains a short β-hairpin composed of two anti-parallel β-strands that is absent in other PDZ domains (Figure 1B) [47]. The NMR structure is consistent with the crystal structure except for minor differences. While both the N- and C-termini are unstructured and flexible in the NMR structure of TIP-1, the C-terminus forms an α-helix in the X-ray structure. Additionally, while the β2–β3 loop region is well-defined in the X-ray structure, it is flexible in the NMR structure.

The three-dimensional structures of TIP-1 bound to ligands that have been deposited to PDB (Protein Data Bank), as of now, have been summarized in Table 1. All of these ligands contain C-terminal recognition motif. The first and only structure-based models of TIP-1 with two different ligands containing internal motif were published in 2012 [39]. These models were calculated through docking studies using experimental constraints from NMR. Thus, there is no structure deposited to PDB of TIP-1 in complex with a ligand having internal motif. However, comparison of available B-factor values from X-ray structures or RMSD (Root Mean Square Deviation) values of NMR structures for both TIP-1 and its complexes (Table 1) shows that the flexibility of the overall protein structure is increased for some complexes while it is reduced for others. It is possible that the flexibility of the overall structure is dependent on a ligand or it could be simply the differences in experimental conditions. Analysis of the average depth of the binding pocket for the structures of the complexes reveals that the presence of W (tryptophan) at the position-5 of the ligand [11] produces comparatively a larger groove for binding (Table 1). It would be interesting to see how the ligands containing internal motifs affect the overall structure and the binding pocket through further structural investigations of TIP-1 interactions with such ligands. It is important to note that the above two observations on the structural flexibility and binding pocket depth of TIP-1 bound to C-terminal recognition motif containing protein can only be verified by additional data.

**Table 1:** Summary of TIP-1 structures deposited in PDB.

PDB ID	Method of Structure Determination	Name of the Ligand ^†^	Sequence of the Ligand	B-Factor (X-ray) /RMSD (NMR) ^‡^	Average Depth of the Binding Pocket (Å) ^§^	References
4E3B	X-ray	iCAL36_L_	ANSRWPTSIL	21.0	23.33	[52]
3SFJ	X-ray	iCAL36	ANSRWPTSII	-	16.42	[51]
2KG2	NMR	No ligand	-	0.473	-	[48]
3DJ1	X-ray	No ligand	-	27.1	-	[47]
3DJ3	X-ray	No ligand	-	37.6	-	[47]
3DIW	X-ray	β-catenin *	NGLAWFDTDL	23.2	19.63	[47]
3GJ9	X-ray	Kir2.3 *	NISYRRESAI	65.8	13.89	[45]
2L4T	NMR	Glutaminase L *	KENLESMV	0.67	14.12	[49]
2L4S	NMR	No ligand	-	0.45	-	[49]
2VZ5	X-ray	No ligand	-	-	-	[46]

^†^ All the ligands have C-terminal motifs. * C-terminal peptides representatives of the proteins used for each study. ^‡^ For X-ray structures, average B-factor values of proteins are given in Å^2^ and for solution NMR structures, RMSD values of well-ordered backbone are given in Å. ^§^ The average depth measurement was obtained from PDBsum database [53,54]; where SURFNET [55] is used to calculate the average depth of the cavities formed within a molecule.

## 5. TIP-1 Recognition of Target Proteins with C-Terminal Recognition Motif

HSQC (Heteronuclear Single Quantum Coherence) spectrum of a protein is regarded as the fingerprint of that protein. Any change in a protein conformation due to ligand binding or change in pH or temperature *etc.* is manifested as perturbations of the chemical shift positions in the HSQC spectrum of the protein. One routine and most effective way to characterize the binding interactions between a protein and its ligand is to monitor the changes in chemical shift positions in an HSQC spectrum of the protein while titrating with increasing concentration of the ligand. The HSQC titration data of TIP-1 with the glutaminase L peptide shows significant perturbations in chemical shift positions for the residues located in the α2 helix [49]. It is clear from the NMR structure of TIP-1-glutaminase L peptide complex that the peptide binds to the groove formed by the α2 helix and the β2 strand of TIP-1 as an antiparallel β-strand through a mechanism known as β-strand addition (Figure 2). However, it does not appear to have many direct interactions with the α2 helix except with His 90, a residue known to play a role in peptide specificity [49]. The significant perturbations in the chemical shift positions of residues in the α2 helix suggest that although there is no direct interaction between the peptide and this helix, the binding is allosterically driven affecting non-interacting residues of the α2 helix [49]. In the NMR time-scale, TIP-1-ligand binding was in the fast exchange regime for all residues except for binding pocket residues containing the ILGF loop (Leu27-Gly35), which were in the intermediate to slow exchange regime. This observation suggests that while some residues experience stronger binding effects, most other residues are allosterically affected [49]. Results of dynamics studies on TIP-1 and TIP-1-glutaminase L peptide complex are consistent with the changes observed in protein backbone RMSD [49]. It is clear from these data that the structural regions of TIP-1 that interact directly with the ligand become rigid. The decrease in flexibility of the binding pocket is compensated by an increase in flexibility distal to the binding site.

**Figure 3:**
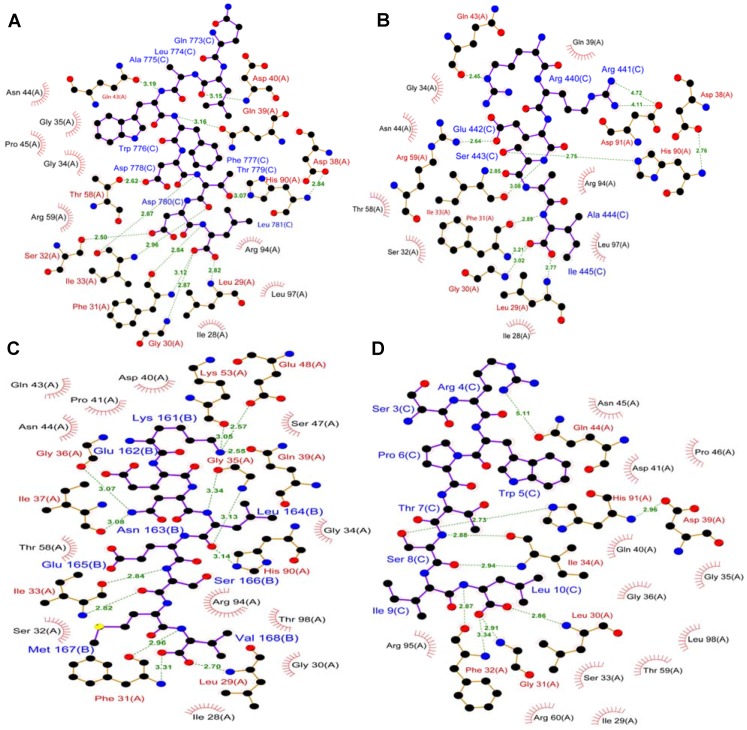
Mechanism of molecular interaction between TIP-1 and ligands. Two-dimensional representations of the interactions of (**A**) C-terminal β-catenin peptide (NQLAWFDTDL-COOH) (PDB code: 3DIW); (**B**) C-terminal Kir 2.3 peptide (RRESAI-COOH) (PDB code: 3GJ9); (**C**) C-terminal glutaminase L peptide (KENLESMV-COOH) (PDB code: 2L4T), (**D**) iCAL36_L_ peptide (ANSRWPTSIL) (PDB code: 4E3B); Residues in this PDB (**D**) were numbered inaccurately. Each residue number should be one less than what is shown. Figures were prepared from LIGPLOT [56] of the interactions in PDBsum database. Hydrogen bonds are represented with green dotted lines labelled with the length of the bond in Å. Dark red “eyelashes” represnt protein residues that make one or more non-bonded contacts to the ligand. Ligand atoms involved in these contacts also carry spokes pointing back. Each residue name and number is followed by the chain ID for the protein or the ligand of respective PDBs. A denotes the protein and B or C denotes the ligand.

The NMR data for the TIP-1-glutaminase L peptide complex suggest that the β2–β3 loop undergoes a conformational change upon ligand binding. This flexible binding loop may add another layer of binding selectivity since it may interact with specific sequences in certain proteins like β-catenin. The TIP-1-β-catenin complex structure shows that the binding pocket is formed by the β1–β2 loop, the β2 strand, the α2 helix and the β2–β3 loop. The last four amino acids of the β-catenin peptide form a β-strand that makes an anti-parallel β-sheet interaction with the β2 strand of TIP-1. The mode of interactions for β-catenin, Kir 2.3 and glutaminase L with TIP-1 is quite similar [49]. Comparison of the structures of these three TIP-1 complexes shows that Leu 29, Gly 30 and Phe 31 residues of the protein, which are part of the ILGF motif, directly interact with the C-terminus of all the above ligands except for glutaminase L peptide where Gly 30 just makes non-bonded contact with the peptide (Figure 3). In other PDZ containing proteins, this ILGF motif is substituted with GLGF motif and has analogous role in the recognition of target proteins. Other TIP-1 residues that play a role in the interactions are from β2 strand, β2-β3 loop, β3 strand, and α2 helix (Figure 3). There are some differences in the residues involved for each ligand. This indicates that while the protein can bind to a wide range of target proteins containing C-terminal recognition motif using a common mode of interaction, there still exist key points of differences in the recognition process to provide specificity. Based on all these observations, it is clear that although there are subtle changes in the protein conformation, the overall structure of TIP-1 is maintained upon binding of a ligand.

## 6. Novel Internal Motif for TIP-1

Phage display and yeast two-hybrid protein interaction library screening identified new targets that are recognized by TIP-1 through interaction with an internal rather than the typical C-terminal PDZ domain recognition motif [39]. Analysis of the peptide sequences obtained through phage display library screening led to the identification of -S/T-X-L/V-D- as a consensus internal binding motif [39]. Several proteins containing this consensus internal motif were identified through protein database searches that were previously shown to be involved in various cancer pathways such as Ankyrin-B, mediator complex subunit 1 (MED1), CYLD cylindromatosis (turban tumor syndrome)/probable ubiquitin carboxyl terminal hydrolase CYLD, MYO18B myosin XVIIIB, autophagy related protein 7 (ATG7), Rho GTPase-activating protein 9 (ARHGAP9), X-ray repair complementing defective repair in CHO cells 4/X-ray repair cross complementing protein 4 (XRCC4), RSF1 remodeling and spacing factor 1, and HIV Tat specific factor 1 [39]. These proteins could be potential targets for TIP-1; although further studies need to be done to establish these proteins as the binding partners of TIP-1 [39]. One of the identified internal recognition motif peptides was reported to suppress the metabolism of human glioblastoma cells in a dose-dependent manner [39]. Thus, the discovery of internal binding motifs for TIP-1 could have a huge impact on the overall understanding of the role of TIP-1 in various cancer pathways.

Very recently, TIP-1 was shown to interact with deltex-1 (DTX1) and Staufen double-stranded RNA binding protein 1 (STAU1), two proteins that lack a C-terminal PDZ domain recognition motif but contain the -S/T-X-L/V-D internal recognition motif. The *in vivo* association of TIP-1 with DTX1 and STAU1 was confirmed in mammalian cells using fluorescence resonance energy transfer (FRET) and confocal microscopy [34].

## 7. TIP-1 Recognition of Internal Motif: Structure-Based Models

Structure-based models of TIP-1 bound to surrogate peptides (ESSVDLLDG and GSGTDLDAS) containing the internal recognition motif have been reported [39]. These models were obtained using experimental distance constraints from NMR studies on the complex of TIP-1 bound to surrogate peptides [39]. Based on the NMR-derived structural models along with the HSQC chemical shift perturbation data, it is clear that the internal motifs bind in the same pocket of the protein as does the canonical C-terminal motif [39]. Although the binding site for both the internal and C-terminal motif is the same, the mode of interaction is quite different [39]. ILGF loop of TIP-1 is flexible in such a way that it could accommodate these two different types of binding motifs [39]. This loop moves in to bind to the smaller terminal carboxylate group (COO^−^) of a C-terminal motif but moves out to accommodate a bulkier side chain carboxylate group (CH2-COO^−^) from residue D of an internal recognition motif [39].

The ILGF motif, the β2 strand, and the α2 helix are the regions of TIP-1 that are most affected upon internal motif binding [39]. Residues LD/VD of the internal motif peptides are reported to be critical for the binding [39]. TIP-1 failed to recognize and consequently did not interact with a double mutant in which LD was mutated to AA. NMR experiments on these mutated internal motif peptides demonstrate the functional importance of residues LD/VD of the partner protein for TIP-1 recognition and binding [39].

## 8. TIP-1 Interaction Network: Significance and Role in Diseases

**Figure 4:**
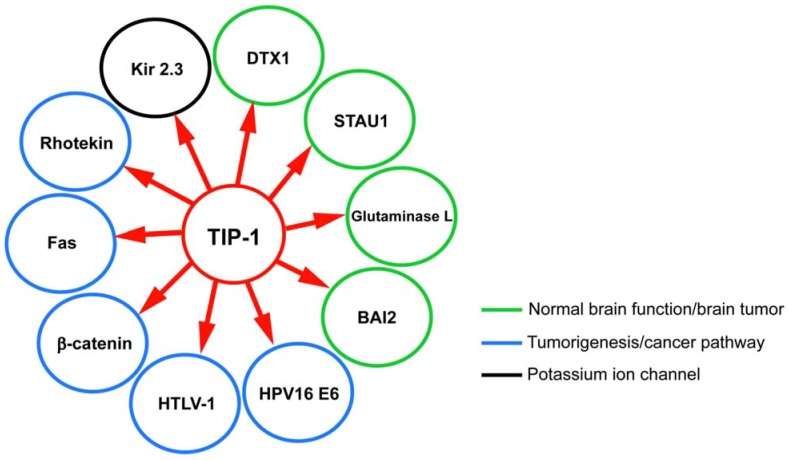
TIP-1 target proteins. Different target proteins of TIP-1 are shown in enclosed circle of specific color to represent pathologically relevant group of proteins. Green circle encloses binding partners that are involved in the normal function of the brain and abnormality in their function could lead to brain tumor. Blue circle encloses target proteins that are involved either directly or indirectly in tumor progression. Black circle encloses protein that is involved in potassium ion transport more tentatively into the cell. All of these partner proteins were shown to interact with TIP-1 through *in vitro* assays (NMR, circular dichroism, fluorescence, X-ray crystallography) and/or through *in vivo* cell based assays or yeast two-hybrid screen.

TIP-1 is a multi-functional protein that interacts with a growing list of target proteins regulating many important signaling pathways involved in cancer and other diseases (Figure 4). The TIP-1 target protein brain-specific angiogenesis inhibitor 2 (BAI2) is expressed primarily in neurons, and is a member of the adhesion G protein-coupled receptors (GPCRs) [35]. Two other TIP-1 target proteins, DTX1 and STAU1, have been implicated in neuronal function [57,58,59]. Furthermore, another TIP-1 target protein involved in the central nervous system is glutaminase L, which is responsible for synaptic transmission and regulation of cerebral concentrations of glutamine and neurotransmitter glutamate [30]. TIP-1 may contribute to the determination of the subcellular distribution and localization of glutaminase and/or regulation of its function [31,60]. Glutaminase L has also been shown to be upregulated in various cancers [61,62,63].

TIP-1 has been reported to be involved in the transcriptional regulation of β-catenin. β-catenin is involved in Wnt signaling pathways and deregulation of the pathway causes various cancerous tumors. TIP-1 overexpression reduces proliferation and anchorage-independent growth of colorectal cancer cells [16]. TIP-1 may have a role in cell apoptosis though its interaction with Fas, which belongs to the TNF receptor family [50,64,65,66]. TIP-1 is also involved in the Rho signaling pathway by interacting with Rho-activator rhotekin [38]. Furthermore, TIP-1 regulates channel expression in the plasma membrane of renal epithelia by interacting with the potassium ion channel Kir 2.3 [33]. It has been shown that TIP-1 modulates the cell transformation by HPV-16 E6 oncoprotein. E6-expressing cells showed increased cell motility and high level of phosphorylated myosin light chains while interacting with TIP-1 and could be inhibited through the silencing of expression of the TIP-1 protein [27,37]. It also recognizes another oncogenic viral protein, HTLV-1 Tax [67]. Other potential TIP-1 internal motif target proteins are involved in various signaling pathways [39].

High level of TIP-1 expression has been seen in human invasive breast cancer cells in athymic mice [28]. Knockdown of TIP-1 resulted in the remarkable down regulation of the genes involved in the cell adhesion and motility in breast cancer cells. TIP-1 can modulate p53 protein stability and is involved in the radio resistance of malignant gliomas. It has been shown to facilitate proliferation of human glioblastoma.

Identifying the interaction network of this protein is a step towards unraveling its involvement in various signaling pathways. However, a detailed biochemical and biophysical investigation is necessary to completely understand the underlying mechanism and the specific role of TIP-1 in these signaling processes. In some cases, TIP-1 inhibits the tumor growth, e.g., inhibition of colon cancer growth through down-regulation of beta-catenin transcriptional activity [16], while in other cases, TIP-1 could aid in the tumor growth such as human glioblastoma [29]. Such differential regulatory activity of TIP-1 could be somewhat explained by the difference in host system and diseased state. While further studies are required to fully comprehend the mechanism of action of TIP-1 in different cellular processes, it may represent a drug target for cancer therapeutics if designed against specific cancer.

## 9. TIP-1 as a Biomarker

Proper and timely diagnosis of a disease such as cancer can help save lives although can be very challenging. For the treatment of cancers and other chronic diseases, it is important to diagnose the disease early enough so that the administered drug regimen could be effective. Thus, discovery of biomarkers for these diseases is extremely important. TIP-1, being involved in many different cancer pathways, has the potential to become a novel biomarker. Recently, it has been shown that in response to ionizing radiation (IR), TIP-1 relocates to the cell plasma membrane surface of both human and mouse lung cancer cells. Such relocation has been linked to the reduced proliferation of tumors and effectiveness of subsequent IR treatment. This is promising for the utilization of TIP-1 as molecular biomarker of tumor response to radiotherapy in a time-efficient manner [68]. Moreover, TIP-1 is also reported as a contributing factor to the tumor-driven angiogenesis in human glioblastoma cell lines in nude mice [29]. It also helps in the activation of Rho GTPases and regulates the growth of glioblastoma [69]. Biochemical analyses using microarrays and antibody arrays revealed that TIP-1 maintains a pro-angiogenic microenvironment within the human glioblastoma cells. Thus, TIP-1 could have prognostic value as well as be a potential therapeutic target for human glioblastoma. Similarly, in other cancer pathways, where overexpression of TIP-1 is associated with the proliferation of tumor cells, TIP-1 could potentially be established as a very important and novel biomarker.

## 10. Conclusions

PDZ domains are one of the most important protein–protein interaction modules in humans and are involved in various signal transduction pathways via their interaction with target proteins. TIP-1 is a multifunctional protein containing a Class I PDZ domain and interacts with several target proteins. Unlike other PDZ domain containing proteins, TIP-1 is composed of a single PDZ domain with a promiscuous binding site. TIP-1 plays an important role in cancer, ion transport, cell polarity and transcription through its interaction with target proteins. Thus, TIP-1 either directly or indirectly regulates various signaling pathways, many of which lead to cancer in humans. TIP-1 has been shown to regulate tumor growth both antagonistically and agonistically depending on the specific cancer in question [16,29]. Thus, besides being a possible target for specific cancer therapeutic intervention, TIP-1 could prove to be a novel biomarker for the prognosis of cancer. However, elucidation of the complete TIP-1 interaction network, significance of these interactions, role in cancer, and the mechanism of the interactions at both the cellular and molecular level will be crucial for rational drug design. The development of both small molecule and peptide-based therapeutics targeting these interactions warrants further studies in this field. Thus, investigation of TIP-1 interactions will have broader application in various aspects of biomedical research and therapeutics.
